# Microshear Bond Strength of Nanoparticle-Incorporated Conventional and Resin-Modified Glass Ionomer to Caries-Affected Dentin

**DOI:** 10.1155/2021/5565556

**Published:** 2021-04-16

**Authors:** Zahra Fattah, Zahra Jowkar, Safoora Rezaeian

**Affiliations:** Oral and Dental Disease Research Center, Department of Operative Dentistry, School of Dentistry, Shiraz University of Medical Sciences, Shiraz, Iran

## Abstract

The purpose of this study was to assess the influence of three different types of nanoparticles (silver (SNPs), titanium dioxide (TNPs), and zinc oxide (ZNPs)) on the microshear bond strength of conventional glass ionomer cement (CGIC) and resin-modified glass ionomer cement based on whether CGIC or RMGIC is used with four subgroups (based on the incorporation of SNPs, ZNPs, and TNPs in addition to a control subgroup) (*n* = 12) as follows: CGIC, CGIC + TNP, CGIC + ZNP, CGIC + SNP, RMGIC, RMGIC + TNP, RMGIC + ZNP, and RMGIC + SNP. After 24 hours, the *μ*SBS of specimens was tested and the obtained data were analyzed using two-way ANOVA and Tukey's HSD test. The obtained results showed that the incorporation of TNPs in two glass ionomers was not statistically significant compared with the control subgroups (*p* > 0.05). In the first group, the highest and lowest mean *μ*SBS were, respectively, observed in the CGIC + SNP subgroup and CGIC + ZNP subgroup. In the second group, RMGIC + ZNP and RMGIC + SNP, respectively, showed the highest and lowest mean *μ*SBS compared to the other subgroups. According to the results, it can be concluded that TNPs can be incorporated into both CGIC and RMGIC without compromising the bond strength of glass ionomers. SNPs and ZNPs can be, respectively, added to CGICs and RMGICs to improve the bond strength of the restoration.

## 1. Introduction

Modern restorative dentistry is mainly focused on preserving tooth structure during cavity preparation and applying nonaggressive methods in treating carious lesions [1]. Carious dentin is composed of two discrete layers: (1) the caries-infected layer which contains irreversible denatured collagen fibrils and is physiologically unremineralizable and (2) the caries-affected dentin (CAD) which is uninfected, partially demineralized, and physiologically remineralizable [2, 3]. Hence, selective caries removal techniques discard the caries-infected layer and maintain the CAD due to its remineralization ability [1, 4, 5]. This approach has increased the demand for remineralizing agents and adhesive restorative materials that bond to the remaining CAD [6].

Glass ionomer cements (GICs) are polymer-based composites with unique advantages including chemical bonding to tooth structure [7], low thermal expansion coefficient [8], translucency [9], and good biocompatibility [8]. Moreover, the gradual fluoride release [8] and cariostatic effects [9] of GICs promote the dental remineralization process. Hence, they have been introduced as promising restorative materials, especially in patients with high caries risk [10].

In recent years, nanomaterials (i.e., materials with at least one dimension below 100 nanometers) have attracted the interest of dental researchers due to their unique characteristics such as their ultrasmall size, large proportion of surface atoms, large surface area, and high surface energy [11]. Among nanomaterials, metal nanoparticles (NPs) such as zinc oxide, silver, and titanium dioxide NPs have been used in dental treatments due to their various advantages such as antibacterial properties and biocompatibility [12, 13].

Zinc oxide NP (ZNP) is an NP with intrinsic antibactericidal activity, biocompatibility, and chemical stability [14–16]. In addition, it has been reported that ZNP has selective toxicity to bacteria with minimum impacts on human cells, motivating its application in dental restorations [17, 18].

Titanium dioxide NP (TNP) is another NP that has shown many advantages in restorative dentistry. The incorporation of TNPs into GICs has resulted in a significant improvement in antibacterial activity [8]. In addition, GICs incorporated with TNPs have biocompatibility with human normal oral cells [19].

Silver NP (SNP) is another metal NP that has gained significant attention, mainly in dentistry, due to its long-term antibacterial properties [20]. The small size of SNPs facilitates their penetration into the cell membranes and changes intracellular processes, leading to increased reactivity and antibacterial activity [20, 21].

Due to the antibacterial properties of metal NPs, it would be beneficial to incorporate them into restorative materials to prevent bacterial colonization at the tooth-restoration interface [22]. However, the mechanical properties of nanoparticle-incorporated restorative materials should also be studied before applying them in CAD treatment. To the best of the authors' knowledge, there is no previous study investigating the effect of incorporating metal NPs into glass ionomer cements on the bond strengths of a conventional glass ionomer cement (CGIC) and a resin-modified glass ionomer cement (RMGIC) to CAD. The null hypothesis of this paper was that incorporating three types of NPs (SNP, TNP, and ZNP) into a CGIC and a RMGIC had no effect on their microshear bond strengths to CAD.

## 2. Materials and Methods

**Figure 1:**
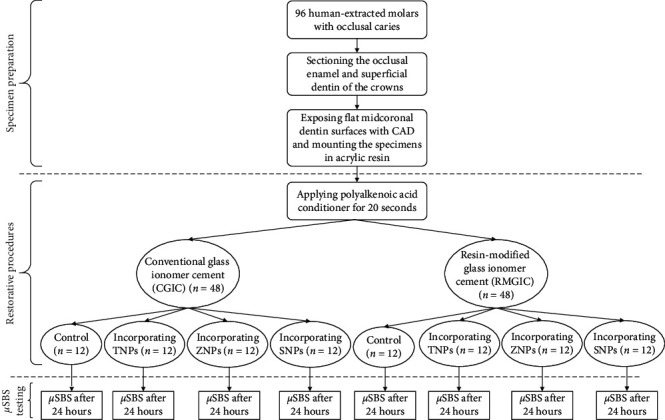
Diagram of the study design. CAD: caries-affected dentin; TNPs: titanium nanoparticles; ZNPs: zinc nanoparticles; SNPs: silicon nanoparticles; *μ*SBS: microshear bond strength.

The diagram of the study design is shown in Figure 1. Different parts of the study will be described in the following.

### 2.1. Specimen Preparation

This experimental study was conducted on 96 human-extracted molars with occlusal caries. The teeth were collected according to the guidelines of the Research Ethics Committee of Shiraz University of Medical Sciences (Protocol # IR.SUMS.DENTAl.REC.1398.49). The teeth were stored in 0.5% chloramine solution at 4°C for no longer than 3 months before use.

The occlusal surfaces of the teeth were prepared by removing the enamel and exposing the dentinal surface using a water-cooled low-speed cutting machine (Mecatome T201 A, Presi, Grenoble, France) at almost the middle one-third of the dentin without exposing the dental pulp. The lesions were stained by flooding the dentine surface with caries-detecting solution (Kuraray Medical Inc., Tokyo, Japan). Then, the occlusal surface that was stained in red was ground flat until reaching the light pink zone [23]. CAD was visually inspected and identified as category 4 according to the North Carolina Dentin Sclerosis Scale. In this category, dentin is glassy in appearance, dark yellow, or even slightly brownish in color, with significant translucency or transparency evident [23].

### 2.2. Restorative Procedures

The teeth were randomly allocated into two groups (*n* = 48) based on the type of the restorative material used (CGIC or RMGIC). Then, the teeth in each group were randomly divided into four subgroups based on the incorporation of three types of NPs (SNPs, ZNPs, and TNPs) (5% wt) into the glass ionomers with an equal number of samples per group (*n* = 12). The nanoparticle powder was weighed carefully using a weighing machine with the accuracy of ±0.0001 g (A&D, GR + 360, Tokyo, Japan). The details of the experimental groups are presented in Table 1.

A uniform smear layer is created on dentin surfaces by slight wet-grinding of surfaces with 600-grit silicon carbide papers for 1 minute. Then, in order to remove any remaining debris, the teeth were rinsed with distilled water. Afterwards, the teeth were mounted in acrylic resin (Acropars, Marlik Co., Tehran, Iran). Prior to restoration placement, a polyalkenoic acid conditioner (Dentin Conditioner, GC Corporation, Tokyo, Japan) was applied on all the samples for 20 seconds with a cotton-tip applicator. Then, the samples were left undisturbed for 10 seconds, water-rinsed for 10 seconds, and gently air-dried for 5 seconds to leave a moist surface. Finally, a polyvinyl chloride microtube with the diameter of 0.7 mm and height of 0.5 mm was placed on each dentin surface. Initially, CGIC and RMGIC powders were separately hand-mixed with nanoparticles and then the obtained powder was placed in amalgam capsules in an amalgamator (Ultramat 2, SDI, Australia) for 20 seconds [24]. Then, the microtubes were filled with CGIC (Fuji II, GC gold label 2, GC International, Japan) and RMGIC (GC Fuji II LC, GC International, Japan). The GIC compositions were prepared according to the instructions of the manufacturer. RMGIC was cured using an LED light-curing unit (VALO, Ultradent Products, South Jordan, UT, USA) at a minimum intensity of 1000 mW/cm^2^ at a distance of 1 mm for 30 s. Before each test, a hand-held radiometer (LED radiometer, Demetron, Kerr, Orange, CA, USA) was used to measure the LED irradiation.

### 2.3. Microshear Bond Strength Testing

All the specimens were stored at 37°C for 24 hours after placement of the restorations. The tubes around the glass ionomer cylinders were removed by gently cutting them using a surgical blade. In order to avoid bias in data collection, blinding was considered during testing the specimens. After that, they were placed in a jig attached to a universal testing machine (Instron, Z020, ZwickRoell, Germany) to measure their microshear bond strengths (*μ*SBS). A knife-shaped indenter applied the shear load to each specimen with a direction parallel to the bonded interface at a crosshead speed of 1 mm/min until failure occurred. The measurements for all the 8 groups were done by one operator at the same time and using the same device.

### 2.4. Failure Mode Analysis

After measuring the bond strength, the failure mode of each fractured specimen was inspected under a stereomicroscope (Carl Zeiss Inc., Oberkochen, Germany) at ×40. The modes of failure were classified as follows: (A) adhesive failure: the failure between the GIC and CAD; (B) cohesive failure: the failure in the GIC; and (C) mixed adhesive: the combination of adhesive failure and cohesive failure.

### 2.5. Statistical Analysis

The normality of the data was checked using the Kolmogorov-Smirnov test. A two-way analysis of variance (ANOVA) model was used to evaluate the effects of the two main factors (the type of the restorative material and the type of the NP incorporated into the glass ionomers). Subgroup analysis was performed using post hoc Tukey's HSD test. All the statistical analyses were performed using SPSS version 17 (SPSS Inc., Chicago, IL, USA). The *p* values less than 0.05 were considered statistically significant.

## 3. Results

The mean and standard deviation of the *μ*SBS of the experimental groups are shown in Table 2. The data showed normal distribution. The two-way ANOVA test showed that all the interaction effects were statistically significant. According to the two-way ANOVA, the type of the incorporated NP significantly affected the *μ*SBS of CGIC and RMGIC. Moreover, the cumulative effect of the material and NP was statistically significant (*p* < 0.001). Hence, the subgroups of each group were compared by the post hoc Tukey's test.

Among the subgroups of group 1 (the group restored with CGIC), the highest mean *μ*SBS was observed in subgroup 4 (CGIC + SNP) (*p* < 0.001), while subgroup 3 (CGIC + ZNP) had a statistically significantly lower mean *μ*SBS than the other subgroups (*p* < 0.001).

Among the subgroups of group 2 (the group restored with RMGIC), subgroup 3 (RMGIC + ZNP) showed a statistically significantly higher mean *μ*SBS than the other subgroups (*p* < 0.05). Moreover, subgroup 4 (RMGIC + SNP) showed the lowest *μ*SBS (*p* < 0.001).

The mean *μ*SBS values of subgroups 2 in both groups (CGIC + TNP and RMGIC + TNP) were not statistically significant compared with the control subgroups for both types of GICs (CGIC and RMGIC) (*p* > 0.05).

According to the failure mode analysis (Table 3), it was observed that adhesive failure was the most prevalent failure mode in all the study groups.

## 4. Discussion

Recently, conservative approaches for caries removal during tooth preparation have been suggested [25]. However, such approaches may leave more carious tissues in the prepared tooth cavity with active bacteria [26, 27]. On the other hand, complete sealing of the tooth-restoration interface is very difficult in practice due to the formation of microgaps at the tooth-restoration interface [28]. This demonstrates the necessity of applying restorative materials with antibacterial properties to prevent the colonization of bacteria at the tooth-restoration interface to preclude recurrent caries [29, 30]. Moreover, compared with sound dentin, CAD has fewer hydroxyapatite crystals and more exposed collagen, which are influential factors in restorative material bonding [31]. Moreover, a caries-affected dentin shows higher porosity, which may lead to the improper infiltration of the restorative material and lower values for the bond strength of the restorative material to CAD [32]. Thus, it would be beneficial to incorporate an antimicrobial agent into the restorative materials. However, it should provide antibacterial properties without negative effects on the bond strength properties of restorative materials [8, 33].

Over the past decades, there has been a growing interest in the application of NPs in restorative dentistry because of their long-term and broad-spectrum antiviral and antibacterial properties [21, 34, 35]. The two main ways to take advantages of the antibacterial properties of NPs in the dental treatments are (1) coating surfaces with NPs to prevent microbial adhesion and (2) incorporation of NPs into dental materials [36, 37]. In this study, the second mechanism was used to benefit from the antibacterial properties of NPs in the restoration process.

The current study investigated the effects of incorporating NPs (TiO_2_, ZnO, and AgO) into a CGIC and a RMGIC on their *μ*SBS to CAD. Previous studies have shown that incorporating low concentrations (ranging from 0.02% to 5%) of NPs into GICs is useful in improving their antibacterial properties without compromising their mechanical and physical properties in addition to their biocompatibility [27, 38, 39]. Therefore, a 5% w/w concentration was selected for the incorporation of three types of NPs into the CGIC and RMGIC to benefit from their antibacterial properties [27, 38, 40].

A good adhesion between the restorative material and the dentinal surface is a determinant aspect for the clinical success of such materials. An important issue, which should be addressed in studying the adhesion of restorative materials, is the bond testing methodology. In this study, *μ*SBS testing was used to evaluate the adhesion of GICs to the tooth structure. Microshear testing allows covering small areas in testing and preparing multiple specimens from a single tooth [41]. In addition, microshear testing does not suffer from the limitations of macroshear testing including the inhomogeneous distribution of stress in the loaded area and the high rate of cohesive failures in the dentinal substrate [42].

### 4.1. TNP

TNPs, which are relatively economical with acceptable mechanical properties and pleasing color, have been previously incorporated into GICs as filler reinforcement agents [8, 33, 43].

In the current study, the *μ*SBSs of the TNP-containing GICs did not show a significant difference from those of their related control subgroups (Table 2). Hence, it can be concluded that TNPs did not negatively affect the chemical bonding abilities of GIC and RMGIC and the curing process of RMGIC. Similar results have been obtained in previous studies conducted on sound dentin [8, 33]. The effects of incorporation of TNPs into a CGIC were investigated in a study by Elsaka et al. [33]. It was shown that this incorporation significantly improved the antibacterial activity of CGIC in addition to its mechanical properties including the compressive strength, flexural strength, and Vickers microhardness without adversely affecting the adhesion to enamel and dentin [33]. According to the obtained results, TNPs can be suggested for incorporation into both types of GICs as they have no adverse effect on the bond strength.

### 4.2. ZNP

Another NP investigated in this study was ZNP. To the best of the authors' knowledge, this is the first study to investigate the effects of incorporating ZNPs on the *μ*SBS of a CGIC and a RMGIC to the caries-affected dentin. According to the results of the present study, adding ZNPs to RMGIC significantly increased *μ*SBS compared to that of the control subgroup. However, the *μ*SBS of the ZNP-containing CGIC was significantly lower than that of the control subgroup. The authors did not find any clear reason for the different *μ*SBSs of CGIC and RMGIC after incorporating ZNPs into them. One possible explanation may be that the incorporation of ZNPs might lead to an adverse effect on the setting reaction of CGICs. However, more studies are required to find the accurate effects of adding ZNPs to CGIC and RMGIC. According to the results obtained in this study, ZNPs can be suggested for incorporation into RMGIC but not into CGIC.

A previous study by Vanajassun et al. investigated the effect of incorporating ZNPs on the shear bond strength of CGIC to sound dentin. They found that the bond strength of CGIC was not affected by the addition of ZNPs, which is in contrast to the result of the present study regarding the negative effect of incorporating ZNP into CGIC on *μ*SBS to CAD [44]. This difference may be related to the different structural properties of sound and caries-affected dentin [3].

There are also some previous works that have focused on assessing the effects of adding ZNPs to dental materials on their antibacterial properties, flexural strength, and hardness [38, 39, 45]. In a study by Garcia et al., it was shown that the antibacterial activity of GIC increased after ZNP incorporation. Moreover, Panahandeh et al. investigated the flexural strength and surface hardness of CGICs and RMGICs supplemented with ZNPs in 2018. It was shown that incorporating ZNPs into GICs did not significantly affect their flexural strengths, while their surface hardness was significantly reduced [45].

### 4.3. SNP

In the current study, the influence of incorporating SNP into GIC on the microshear bond strength to CAD was evaluated. The obtained results showed that the SNP-containing CGIC provided a significantly higher *μ*SBS compared to the control group. Recent studies have suggested that NPs incorporated into GIC fill the voids in the cement matrix formed due to the trapped air during mixing the cement [13, 33]. The increased *μ*SBS of the SNP-containing GIC may be justified by the nanometer sizes of the SNPs added to GIC and the probable improved packing of NPs within the set cement matrix. Moreover, the incorporation of SNP into GIC may increase the range of particle size distribution and, hence, these NPs may fill the empty spaces between the larger glass particles. This may provide an extra bonding site for the polyacrylic polymer and, in turn, reinforce the GIC [46, 47].

This study showed the positive effect of SNP incorporation into CGIC, which was similar to a previous study by Jowkar et al. [48]. The bond strength improvement in the previous studies was justified by microscopic observations, showing the reduced air voids and microcracks in the set matrix of the GIC due to its increased homogeneity and improved consistency after the incorporation of silver NPs [13]. This explanation also justifies the findings of the current study regarding the improvement of the *μ*SBS of SNP-incorporated CGIC compared to that of the control subgroup to the CAD. The results obtained in this study showed that the incorporation of SNP does not adversely affect the chemical bonding ability of CGIC to dentin; meanwhile, it may have a positive influence on its bond strength values.

In the present study, the mean *μ*SBS of the SNP-containing RMGIC to dentin was decreased compared to that of the control subgroup. Discoloration and color change to a tone of gray are the common problems of SNPs [37]. This may increase the opacity of the RMGIC and its incomplete curing, which may decrease the bond strength of RMGIC to the CAD. Hence, it is not recommended to incorporate SNPs into RMGIC due to their interference with the curing process.

In this study, SNP and ZNP showed agnostic effects; that is, applying ZNP increased *μ*SBS in RMGIC, while decreasing it in CGIC, and the SNP decreased the bond strength in RMGIC but increased it in CGIC. This finding might be due to the possible altered surface energy and wettability of the dentin substrate after applying nanoparticle, which lead to their different interactions with the tooth structure and the restorative material. Moreover, other factors such as possible variations in the size and surface-to-volume ratio of the applied nanoparticles may affect the results of the research which can be assessed by conducting more studies on the bond strength properties of the nanoparticles to the tooth structure.

The current study had some limitations. In addition, since both sound dentin and caries-affected dentin provide bonding areas in clinical situations, future studies can be conducted to compare sound and caries-affected dentin considering various NPs and GICs. There are also some other limitations with regard to clinical situations. The effects of mechanical aging or thermal cycling on the specimens were not studied. Future investigations can focus on the impacts of temperature changes and intermittent/complex functional forces from various directions in the oral environment. Since this study was an in vitro study, its results should be confirmed in the future in in vivo studies. Moreover, it would be interesting to investigate more issues in future studies including the long-term bond strength properties, the antibacterial and anticaries effects of the NPs, and the probable release of NPs into the oral cavity and saliva.

## 5. Conclusions

Considering the limitations of this study, the following can be concluded:

1. The microshear bond strengths of both CGIC and RMGIC were not affected by the incorporation of TNPs. Therefore, TNPs can be used due to their potential antibacterial activities without compromising the bond strength of glass ionomers.
2. The *μ*SBS was increased after incorporating the SNPs of CGIC to CAD, while ZNP incorporation decreased the *μ*SBS. Hence, SNPs can be added to CGICs to improve the bond strength of the restoration and to provide antibacterial properties.

## Figures and Tables

**Table 1:** The details of the experimental groups.

Group	Subgroup	GI type	The incorporated nanoparticle	Abbreviation	Description
1	1	Conventional glass ionomer cement	—	CGIC	Conventional GIC control without intervention
	2	Conventional glass ionomer cement	Titanium dioxide nanoparticle	CGIC + TNP	Conventional GIC incorporated with TNPs^1^ at 5% (w/w)
	3	Conventional glass ionomer cement	Zinc oxide nanoparticle	CGIC + ZNP	Conventional GIC incorporated with TNPs^2^ at 5% (w/w)
	4	Conventional glass ionomer cement	Silver nanoparticle	CGIC + SNP	Conventional GIC incorporated with TNPs^3^ at 5% (w/w)

2	1	Resin-modified glass ionomer cement	—	RAMGIC	Resin-modified GIC control without intervention
	2	Resin-modified glass ionomer cement	Titanium dioxide nanoparticle	RAMGIC + TNP	Resin-modified GIC incorporated with TNPs at 5% (w/w)
	3	Resin-modified glass ionomer cement	Zinc oxide nanoparticle	RAMGIC + ZNP	Resin-modified GIC incorporated with ZNPs at 5% (w/w)
	4	Resin-modified glass ionomer cement	Silver nanoparticle	RMGIC + SNP	Resin-modified GIC incorporated with SNPs at 5% (w/w)

^1^Titanium dioxide NPs. ^2^Zinc oxide NPS. ^3^Silver NPs.

**Table 2:** Mean (standard deviation) of the microshear bond strength (*μ*SBS, MPa) in each experimental group.

	Mean ± SD			
	Control	TNP	ZNP	SNP
CGIC	3.77 ± 0.89 B, a	4.15 ± 0.83 B, a	2.03 ± 0.39 C, a	6.96 ± 1.58 A, a
RMGIC	8.12 ± 1.83 B, b	7.27 ± 0.98 B, b	10.15 ± 2.06 A, b	3.48 ± 0.66 C, b

The mean values followed by the same uppercase letter indicate no significant statistical difference in the row and the same lowercase letter indicates no significant statistical difference in the column (*p* > 0.05).

**Table 3:** Results of failure mode analysis.

Groups	Subgroups	Experimental condition	Failure mode		
			Adhesive	Cohesive	Mixed
1	1	CGIC	1	0	11
	2	CGIC + TNP	2	0	10
	3	CGIC + ZNP	2	0	10
	4	CGIC + SNP	3	0	9

2	1	RMGIC	2	0	10
	2	RMGIC + TNP	4	0	8
	3	RMGIC + ZNP	2	0	10
	4	RMGIC + SNP	3	0	9

## Data Availability

The data that support the findings of this study are available on request from the corresponding author.
